# Improved secondary caries resistance via augmented pressure displacement of antibacterial adhesive

**DOI:** 10.1038/srep22269

**Published:** 2016-03-01

**Authors:** Wei Zhou, Li-na Niu, Li Huang, Ming Fang, Gang Chang, Li-juan Shen, Franklin R. Tay, Ji-hua Chen

**Affiliations:** 1 State Key Laboratory of Military Stomatology, Department of Prosthodontics, School of Stomatology, The Fourth Military Medical University, Xi’an, Shaanxi, China; 2 State Key Laboratory of Military Stomatology, Department of General Dentistry and Emergency, School of Stomatology, The Fourth Military Medical University, Xi’an, Shaanxi, China; 3 Department of Endodontics, College of Dental Medicine, Augusta University, Augusta, Georgia, USA

## Abstract

The present *in vitro* study evaluated the secondary caries resistance potential of acid-etched human coronal dentin bonded using augmented pressure adhesive displacement in conjunction with an experimental antibacterial adhesive. One hundred and twenty class I cavities were restored with a commercial non-antibacterial etch-and-rinse adhesive (N) or an experimental antibacterial adhesive (A) which was displaced by gentle air-blow (G) or augmented pressure air-blow (H). After bonding and restoration with resin composite, the resulted 4 groups (N-G, N-H, A-G and A-H) were exposed to *Streptococcus mutans* biofilm for 4, 8, 15, 20 or 25 days. The development of secondary caries in the bonding interface was then examined by confocal laser scanning microscopy (CLSM) and scanning electron microscopy (SEM). Data acquired from 15, 20 and 25 days of artificial caries induction were analyzed with three-way ANOVA at α = 0.05. The depth of the artificial carious lesions was significantly affected by “adhesive type” (Single Bond 2 *vs* experimental antibacterial adhesive p = 0.003), “intensity of adhesive displacement” (gentle *vs* augmented-pressure adhesive displacement; p < 0.001), as well as “artificial caries induction time” (p < 0.001). The combined use of augmented pressure adhesive displacement and experimental antibacterial adhesive reduces the progression of secondary caries.

Secondary caries is one of the most important causes of failure of bonded restorations. In the oral environment, the resin-dentin interface is under constant challenge by factors such as water, enzymes, masticatory forces, temperature and microbes derived from plaque biofilms and becomes unstable^1^,^2^. Leakage and gaps that form in this weakened interface provide a pathway for invasion of oral plaque biofilms and development of secondary caries around the tooth-restorative margins^3^. Thus, improvement of bond durability and prevention of bacterial ingress are pivotal issues to be considered for reducing the rate of secondary caries.

Reasons for degradation of resin-dentin bonds include resin hydrolysis, poor infiltration of resin monomers into acid-etched dentin and subsequent degradation of the demineralized collagen by activation of endogenous proteases such as matrix metalloproteinases and cysteine cathepsins trapped within the mineralised collagen matrix^2^,^4^. A recent study reported the use of 0.3 MPa air-spray pressure (compared to the use of conventional 0.1 MPa air-spray) to displace adhesives after their application with a moist bonding technique, for improving resin infiltration into the acid-etched dentin^5^. The use of the augmented pressure adhesive displacement strategy resulted in more homogeneous resin tags that exhibited more lateral ramifications. Because nanoleakage was rarely observed along the bonding interface when infiltrated etch-and-rinse adhesives were displaced using the augmented pressure adhesive displacement strategy, the authors opined that the strategy may improve dentin bonding durability and enhance the ability of the resin-dentin interface to resist bacteria ingress and destruction of the tooth structure. The hypothesis that the adjunctive use of the augmented pressure adhesive displacement strategy during bonding with etch-and rinse adhesives produces resin-dentin interfaces with better caries-resistance requires further *in vitro* substantiation prior to proof-of concept validation with animal trials.

Antibacterial adhesives and resin composites have been developed for protecting tooth and restorations from attack by oral plaque biofilms. Polymerizable quaternary ammonium salts (QAS) have been shown to be excellent antibacterial materials for incorporation into dentin adhesives^6^,^7^. The authors have developed a series of QAS monomers, which, when incorporated into restorative materials, endow the materials with contact inhibition antibacterial properties without adversely affecting their mechanical properties^8^,^9^,^10^. Nevertheless, the antibacterial potential of adhesives containing novel QAS monomers has to be evaluated using more clinically relevant models.

Accordingly, the objective of the present study was to evaluate the secondary caries resistance potential of acid-etched dentin bonded using augmented pressure adhesive displacement in conjunction with an experimental antibacterial adhesive. An invasive model was used for examining the development of secondary caries by confocal laser scanning microscopy (CLSM) at different time periods. Artificial carious lesions detected by autofluorescence in the CLSM images were physically confirmed using scanning electron microscopy (SEM). The null hypotheses tested were: 1) the intensity of air pressure applied for displacement of adhesives applied to moist acid-etched dentin has no effect on the ability of the resin-dentin interface to resist demineralization by a single-species acidogenic bacteria biofilm; 2) the use of an antibacterial adhesive has no effect on the ability of the resin-dentin interface to resist demineralization by the single-species acidogenic bacteria biofilm; and 3) the combined use of augmented pressure adhesive displacement and antibacterial adhesive does not produce better caries resistance when compared with the separate use of those modalities.

## Materials and Methods

### Specimen preparation

The studies were carried out in accordance with the protocol approved by the Ethics Committee for Human Studies of the Fourth Military Medical University. Sixty-six caries-free human third molars were collected after obtaining the patients’ informed consent, stored at 4 °C and used within 1 week after extraction. Occlusal enamel was removed perpendicular to the longitudinal axis of each tooth using a slow-speed diamond saw (MTI Co., Shenyang, China) with water cooling. A second cut was made 2 mm below the previous cut to obtain a 2 mm-thick dentin disk. Two class I cavities (4 mm × 2 mm and 1 mm deep) were prepared with tungsten carbide burs in a single dentin disk for comparison of conventional vs augmented pressure adhesive displacement. Each dentin disk was attached with cyanoacrylate glue (Zapit, Dental Ventures of America, Anaheim Hills, CA, USA) to a Plexiglas platform assembly to deliver 20 cm of water pressure during bonding^11^.

**Figure 1:**
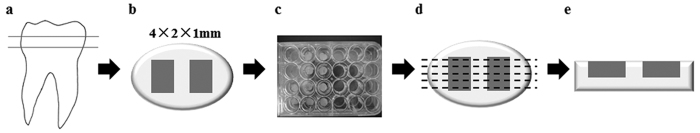
Specimen preparation. (**a**) Occlusal enamel and roots removed to obtain a 2-mm thick dentin disk; (**b**) Two class I cavities (width × length × depth was 4 × 2 × 1 mm) were prepared in the dentin disk for different bonding treatments; (**c**) Sterile specimens were placed into *S. mutans* emulsions for induction of artificial caries; (**d**) Specimens were removed from bacteria emulsion and sectioned into slices after designated periods of artificial caries induction; (**e**) 0.5-mm thick specimen slices were rinsed to remove loosely-attached biofilms for CLSM and SEM.

The materials employed were: 1) a 2-step etch-and-rinse adhesive (Single Bond 2, 3 M ESPE, St. Paul, MN, USA); 2) an experimental antibacterial adhesive, prepared by adding 10 wt% 2-methacryloxylethyl dodecyl methyl ammonium bromide (MAE-DB) into Single Bond 2. This is the highest concentration which showed strong bactericidal activities without compromising the mechanical properties of the resin material^9^; 3) a resin composite (Filtek^TM^ Z250, 3 M ESPE). Each cavity was treated by 37% phosphoric acid gel (Scotchbond™ Universal Etchant, 3 M ESPE) for 15 sec, blot-dried and moist-bonded with the designated adhesive (Table 1). Adhesive evaporation was performed using one of the two designated adhesive displacement strategies (Table 1). This resulted in 4 groups (n = 15) that were designated as N-G, N-H, A-G and A-H, respectively (Fig. 1) according to the type of adhesive employed (N: commercial non-antibacterial adhesive; A: experimental antibacterial adhesive) and the adhesive displacement strategy employed (gentle air-blow using 0.1 MPa air pressure for 5 sec; augmented pressure air-blow using 0.3 MPa air pressure for 5 sec). For each tooth, one cavity was filled using an adhesive with gentle adhesive displacement, while the other cavity was filled using the same adhesive using augmented pressure adhesive displacement.

After bonding and restoration with resin composite, the surface of each disk was polished with silicon carbide abrasive papers of increasing fineness, up to 2000 grit, and sonicated for 5 min with distilled water. All surfaces of each dentin disk were coated twice with nail varnish except for the filled surface.

### Artificial caries

*Streptococcus mutans* (ATCC 25175, American Type Culture Collection, Manassas, VA, USA) was used to create a single-species acidogenic bacterial biofilm for generating artificial secondary caries. The bacteria were pre-cultured anaerobically in brain heart infusion (BHI) broth (Hopebio, Qing Dao, China) at 37 °C for 24 hr, using 5% CO_2_ atmosphere. For the experiments, the bacteria suspension was diluted to 10^7 ^CFU/mL in BHI broth supplemented with 1% sucrose^12^.

Before co-culturing with bacteria, all polished specimens (60 dentin disks) were sterilized by exposure to ultraviolet light for 4 hr. Each specimen was placed inside one well of a sterile 24-well plate. Freshly prepared *S. mutans* suspension (1.5 mL) was then added to each well. The bacterial suspensions were incubated in an anaerobic work station (Whitley H85 Hypoxystation, Shipley, West Yorkshire, BD17 7SE, United Kingdom) and the growth medium was replenished every two days. The pH values of the bacterial suspensions were recorded with a pH meter every two days to evaluate bacterial activity. Specimens were removed from bacterial suspensions after 4, 8, 15, 20 or 25 days (n = 6 cavities for each designated time period) to evaluate the extent of artificial caries formation along the dentin-restorative margins.

### Confocal laser scanning microscopy (CLSM)

On retrieval, the specimens were sonicated in distilled water to remove as much of the attached bacterial biofilm as possible. Each bonded dentin disk was sectioned into slices in a direction parallel to the longitudinal axis of the tooth, using the slow-speed diamond saw under water cooling. Both sides of each slice was serially-polished with 400, 800, 1200 and 2000 grit silicon carbide papers and sonicated with distilled water. Four 0.5 mm thick slices were obtained from one dentin disk. Each slice was placed on a microscopic glass slide, covered with ethylene glycol to prevent water evaporation^13^,^14^,^15^, and examined wet using CLSM (FV1000, Olympus Corp., Tokyo, Japan). The excitation wavelength was 488 nm and a 505 nm long-pass filter was used for detecting the autofluorescence emitted by the artificial carious dentin^16^,^17^,^18^,^19^,^20^. Scanning thickness was 5 μm for all the specimens. Images were obtained using FV10-ASW 3.1 Viewer (Olympus Corp., Tokyo, Japan). The depth of an artificial carious lesion was measured from the top of resin composite to the lowest level of autofluorescence exhibited by the carious lesion.

### Statistical analyses

Because no carious lesions were detected from all groups at 4 and 8 days, only data obtained at 15, 20 and 25 days were used for statistical analyses. For each group (N-G, N-H, A-G and A-H) at each designated time period (15, 20 and 25 days), the use of 3 dentin disks with 4 slices per disk and 2 restorations per slice resulted in 24 measurements per group-time period. Because the 4 slices were taken from the same tooth, the mean of the data obtained from the 4 slices of each restoration was used to represent the mean lesion depth of each restoration, with “cavity” as the statistical unit. This resulted in 3 dentin slices × 2 cavities (n = 6) for each group-time period for statistical analysis. Data were analyzed with three-factor analysis of variance (ANOVA) to examine the effects of “adhesive type”, “intensity of adhesive displacement” and “artificial caries induction time”, and the interaction of these factors on the depth of the artificial carious lesions. Parametric statistical methods were employed after affirming that the normality (Shapiro-Wilk test; p = 0.512) and equal variance assumptions (modified Levene test; p = 0.350) of the data had not been violated. Post-hoc pairwise comparisons were examined using the Holm-Sidak method. For all analyses, statistical significance was preset at α = 0.05.

To further determine if significant differences were present among the four groups (N-G, N-H, A-G, A-H) in each time period, data sets from each of the three time periods (15, 20 or 25 days) were analyzed separately by one-factor ANOVA at α = 0.05. For post-hoc comparisons, Bonferroni adjustment of the alpha value (critical α: 0.05/3 = 0.0167) was used to control the familywise error rate and reduce the chances of obtaining false-positive results (type I errors) in the Holm-Sidak multiple comparison procedures^21^.

### CLSM of non-carious, unetched and acid-etched dentin

Six additional dentin disks were prepared in the manner previously described, to investigate whether the intense autofluorescence exhibited by non-carious resin-dentin interfaces was originated from acid-etched dentin, unetched mineralized dentin or from the adhesive. Three kinds of treatment were performed (n = 2): 1) each dentin surface was etched with 15% phosphoric acid for 15 sec, moist-bonded with a thick layer of Single Bond 2, light-cured, covered with Z250 resin composite and light-cured; 2) each dentin surface was for 15 sec, covered with Z250 without adhesive application, and light-cured; 3) Single Bond 2 was directly applied on each unetched dentin surface, light-cured, covered with Z250 and light-cured. After bonding, each specimen was sectioned into slices and observed with CLSM for identifying the autofluorescence emitted by different components of the resin-dentin interface, using the protocol described above.

### Scanning electron microscopy (SEM)

After CLSM, each specimen surface was etched with 15% phosphoric acid for 10 sec er to bring the polished surface into relief. Dehydrated specimen was mounted on aluminum stubs, sputter-coated with gold/palladium and examined by SEM (Hitachi FE-SEM 4800, Tokyo, Japan) at 5 kV. The artificial caries generated after 20 days induction along the restoration-dentin interface was confirmed with energy dispersive X-ray analysis (EDS) (PV72-45030LC, Ametek, USA) examination at 30 kV.

## Results

Elemental map-scans of a representative artificial caries generated along the restoration-dentin interface in N-G group after 20 days induction are presented in Supplementary S1. The lower Ca and P content and higher C and O content in surface layer of the specimen indicate demineralization of this area.

**Figure 2:**
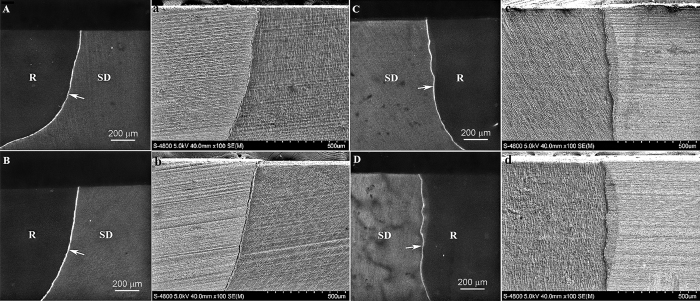
CLSM and supportive SEM images of representative bonding interfaces derived from the different groups after 4 days of artificial caries induction. (**A**) CLSM image of N-G group; (a) SEM image of N-G group; (**B**) CLSM image of N-H group; (b) SEM image of N-H group; (**C**) CLSM image of A-G group; (c) SEM image of A-G group; (**D**) CLSM image of A-H group; (d) SEM image of A-H group. No dentin surface demineralization or artificial caries could be identified from the restorative margin in both CLSM and SEM for all groups, as indicated by the absence of autofluorescence along the dentin surface. The non-carious resin-dentin interface exhibited intense autofluorescence and was continuous from surface to bottom of the restorative interface (arrows). The resin composite (R) did not emit autofluorescence, while faint autofluorescence was emitted by the mineralized sound dentin (SD). Similar features (i.e. no dentin surface demineralization or artificial caries along the restorative margin) were observed in all species derived from the different groups after 8 days of artificial caries induction (Supplementary S2).

**Figure 3:**
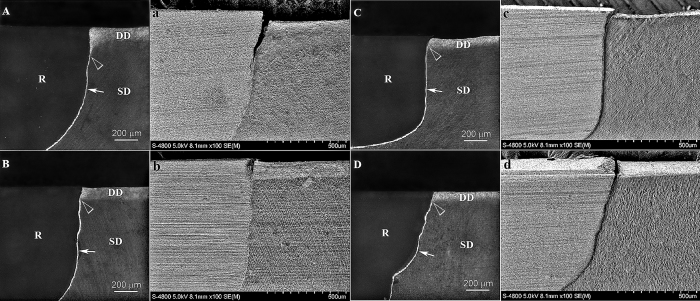
CLSM and supportive SEM images of representative bonding interfaces from the different groups after 20 days of artificial caries induction. (**A**) CLSM image of N-G group; (a) SEM image of N-G group; (**B**) CLSM image of N-H group; (b) SEM image of N-H group; (**C**) CLSM image of A-G group; (c) SEM image of A-G group; (**D**) CLSM image of A-H group; (d) SEM image of A-H group. Similar to specimens obtained from all the other caries induction time periods, intense autofluorescence was emitted by the resin-dentin interface (arrows) but not from the resin composite (R). Faint autofluorescence was emitted by the mineralized sound dentin (SD). Demineralization of dentin by acidogenic plaque biofilm can be identified as a layer of autofluorescence along the dentin surface (DD) in the CLSM images. Artificial caries formation can be recognized as a continuation of the autofluorescence along the superficial part of the resin-dentin interface (open arrowheads). Depending on the severity of the artificial caries, a wedge-shaped lesion is often formed in groups with more extensive acid penetration along the resin-dentin interface (e.g. group N–G). Unlike CLSM imaging, features of dehydration shrinkage of the demineralized dentin surface collage and crack formation along the cavosurface margin could be seen in all SEM specimens. Similar features (i.e. dentin surface demineralization or artificial caries along the restorative margin) were observed in all species derived from the different groups after 15 or 25 days of artificial caries induction (Supplementary S3 and S4), with variations in the thickness of dentin surface demineralisation and depth of artificial caries formation.

Representative CLSM and SEM images of artificial caries generated along the restoration-dentin interface after 4, 8, 15, 20 or 25 days of artificial caries induction are shown in Figs 2 and 3. A unique, previously unreported feature that is characteristic of all CLSM images is the appearance of intense autofluorescence along the resin-dentin interface in the entire cavity. The resin composite restoration did not emit any noticeable autofluorescence, while faint autofluorescence was emitted by the mineralized dentin. The occurrence of this intense interfacial autofluorescence is independent of the milder form of autofluorescence produced by bacteria-induced artificial caries on the dentin surface.

No artificial caries could be detected from the CLSM images of all specimens after 4 days (Fig. 2; CLSM images labeled with upper case letters) or 8 days (Supplementary S2; features similar to Fig. 2) of artificial caries induction. After 15 days, artificial secondary caries could be seen in all groups (Supplementary S3; features similar to Fig. 3), which was manifested in CLSM images as a wedge-shaped region of autofluorescence between the dentin and resin composite. Demineralization of the dentin surface by acid produced by *S. mutans* biofilms resulted in the emittance of a surface band of autofluorescence that was continuous with the wedge-shaped lesion. The depth of the artificial carious lesions increased progressively when the artificial caries induction time was increased to 20 days (Fig. 3; CLSM images labeled with upper case letters) and 25 days (Supplementary S4; features similar to Fig. 3). Artificial caries that emitted autofluorescence in CLSM imaging corresponded to zones of shrunken, partially-demineralized dentin when the specimens were examined by SEM (Figs 2, images labeled with lower case letters). Because of the dehydration and SEM examination in high vacuum, artifactual cracks and shrinkage of the biofilm-demineralized collagen matrix were ubiquitous along the cavosurface margins and dentin surfaces. These dehydration artifacts were absent when the specimens were covered with ethylene glycol to prevent water evaporation during CLSM imaging.

Three-factor ANOVA indicated that the depth of the artificial carious lesions was significantly affected by “adhesive type” (Single Bond 2 *vs* experimental antibacterial adhesive p = 0.003), “intensity of adhesive displacement” (gentle *vs* augmented-pressure adhesive displacement; p < 0.001), as well as “artificial caries induction time” (p < 0.001). Of the four possible interactions among the three factors, only the interaction between “intensity of adhesive displacement” and “artificial caries induction time” was significant (p = 0.013). For the factor “artificial caries induction time”, Holm-Sidak pairwise comparisons indicated that significant differences were present between the 25 days and 15 days results (p < 0.001), and between the 25 days and 20 days results (p < 0.001). There was no significant difference between the 15 days and 20 days results (p = 0.091).

For comparisons of the factor “intensity of adhesive displacement” within “adhesive type”, the use of gentle adhesive displacement generated significantly deeper artificial lesions than the use of augmented pressure adhesive displacement in acid-etched dentin bonded with Single Bond 2 (p < 0.001) or the experimental antibacterial adhesive (p = 0.003). For comparisons of the factor “adhesive type” within “intensity of adhesive displacement”, artificial lesion depth was significantly deeper in cavities bonded with Single Bond 2, compared with the experimental adhesive, when gentle air-blow was used for adhesive displacement (p = 0.009). Conversely, there was no difference between Single Bond 2 and the experimental adhesive when augmented pressure was employed for adhesive displacement (p = 0.088).

For comparisons of the factor “artificial caries induction time” within cavities bonded with Single Bond 2, significant differences in lesion depth were observed between the 25 days and 15 days results (p < 0.001) and between the 25 days and 20 days results (p < 0.001); results from 15 days were not significantly different from those obtained after 20 days of artificial caries induction (p = 0.203). The same trend was identified in cavities bonded with the experimental antibacterial adhesive: significant differences in lesion depth were observed between the 25 days and 15 days results (p < 0.001) and between the 25 days and 20 days results (p < 0.001), while there was no significant difference between the results obtained at 15 days and 20 days (p = 0.254). For comparisons of the factor “adhesive type” within “artificial caries induction time”, significant difference in lesion depth was observed between cavities bonded with the two adhesives at 15 days (p = 0.038) and 20 days (p = 0.048), but not at 25 days (p = 0.202).

For comparisons of the factor “artificial caries induction time” within cavities bonded using gentle adhesive displacement, significant differences in lesion depth were observed between the 25 days and 15 days results (p < 0.001), between the 25 days and 20 days results (p < 0.001), but not between the 15 days and 20 days results (p = 0.087). The same trend was identified in cavities bonded using augmented pressure adhesive displacement: significant differences in lesion depth were observed between the 25 days and 15 days results (p < 0.001), between the 25 days and 20 days results (p < 0.001), but not between the 15 days and 20 days results (p = 0.477). For comparisons of the factor “intensity of adhesive displacement” within “artificial caries induction time”, no significant difference in lesion depth could be identified at 15 days from cavities bonded using gentle or augmented pressure for adhesive displacement (p = 0.178). In contrast, significant difference in lesion depth were detected in cavities bonded with the two adhesive displacement strategies at 20 days (p = 0.035) and 25 days (p < 0.001).

**Figure 4:**
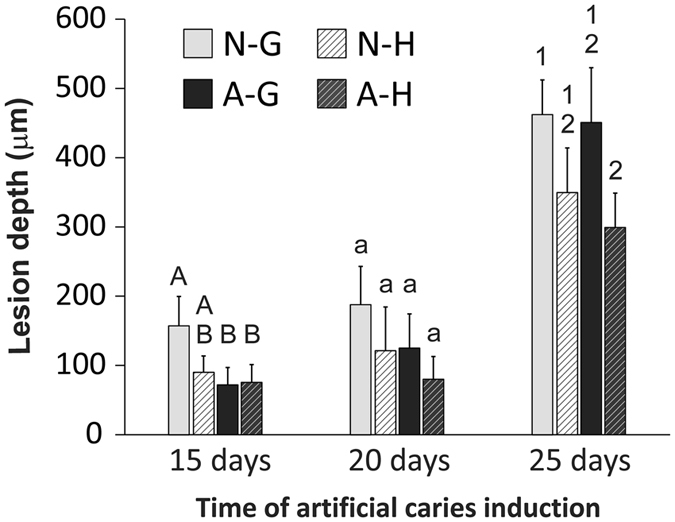
Bar chart showing variation in lesion depths (values represent means and standard deviations) among the four experimental groups after artificial caries induction for 15, 20 and 25 days and the results of post-hoc pairwise comparisons among the four groups. For each designated time period, groups labeled with the same designators (upper case letters for 15 days, lower case letters for 20 days, numerals for 25 days) are not significantly different (p > 0.0167).

Results of post-hoc pairwise comparisons on lesion depth in the four experimental groups at each of the three designated time periods are shown in Fig. 4. After artificial caries induction for 15 days, significant difference was identified among the four groups (one-factor ANOVA; p = 0.002). Pairwise comparisons, with Bonferroni adjusted critical α value to determine the significant level, indicated that lesion depth in group N-G was significantly higher than those in A-H (p = 0.004) and A-G (p = 0.004), while all other comparisons were not significantly different (p > 0.0167). After artificial caries induction for 20 days, no significant difference in carious lesion depth could be detected among the four groups (one-factor ANOVA; p = 0.108). Significant difference was observed among the four groups after artificial caries induction for 25 days (one-factor ANOVA; p = 0.004). Lesion depth was significant higher in group N-G compared with group A-H (p = 0.009), with no significant differences observed in the other pairwise comparisons (p > 0.0167).

**Figure 5:**
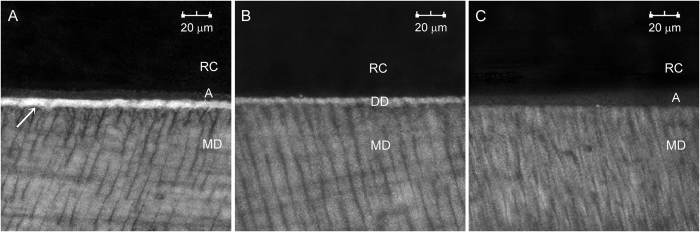
CLSM images of flat dentin surfaces for identifying the intensity of autofluorescence emitted by different components of the non-carious resin-dentin interface. (**A**) Single Bond 2-bonded acid-etched dentin. (**B**) Acid-etched dentin without adhesive application. (**C**) Application of Single Bond 2 on unetched dentin. RC: resin composite; (**A**) adhesive; DD: demineralized dentin; Arrow: hybrid layer; MD: mineralized dentin.

The intensity of autofluorescence emitted by different restorative and dentin components are shown in Fig. 5. When acid-etched dentin was bonded with Single Bond 2, faint autofluorescence was emitted by the adhesive and the mineralized dentin, while the hybrid layer emitted strong autofluorescence (Fig. 5A) that was stronger than that emitted by the artificial carious dentin (Figs 2 and 3). Autofluorescence emitted by unbonded acid-etched dentin was slightly stronger than that emitted by the underlying mineralized dentin (Fig. 5B), but was not as intense that emitted by the hybrid layer in bonded dentin. Autofluorescence emitted by polymerized Single Bond 2 was, in turn, fainter than that emitted by the unetched mineralized dentin (Fig. 5C). Despite the presence of zirconia/-silica fillers, no autofluorescence could be detected from the hybrid resin composite.

## Discussion

The most pressing concern in the discussion of the augmented pressure adhesive displacement is whether the strategy can be translated into a clinically-feasible technique for bonding of etch-and-rinse to deep, vital dentin with small remaining dentin thickness, without adversely causing post-operative hypersensitivity^22^ and/or displacement of the odontoblast nuclei and intracellular contents such as mitochondria into the dentinal tubules^23^,^24^. Based on the hydrodynamic theory^25^, application of strong air blasts to vital tooth cavities results in evaporation of the water component of the dentinal fluid^26^. Creation of a negative capillary pressure, in turn, causes rapid outward fluid flow that carries the intracellular contents of the odontoblasts into the dentinal tubules, as well as stimulates the Aδ nerve fibres in the vicinity of the odontoblasts to elicit a pain response^27^. In Dr. Brännström’s own words: “the aspiration of odontoblasts into the dentinal tubules as an immediate effect of physical stimuli applied to exposed dentin seems to result from loss of substance at the distal apertures of the tubules and subsequent outward flow of the tubular contents through capillary action”^24^.

For the augmented pressure adhesive displacement strategy, patent dentinal tubules are first filled with a dental adhesive prior to the application of strong air-blow. Any “loss of substance” would only be the removal of adhesive solvent, the process of which renders the “distal apertures of the tubules” densely-filled with more saturated and viscous adhesive co-monomers. Thus, the augmented pressure adhesive displacement strategy is unlikely to create outward fluid movement that is rapid enough to cause displacement of odontoblast contents into the dentinal tubules, or to stimulate the Aδ nerve fibres to elicit post-operative hypersensitivity. It should be emphasized that slow outward fluid movement of water can still occur through the uncured, hydrophilic and hyperosmotic adhesive within the dentinal tubules. However, slow outward fluid across the resin-dentin interface occurs routinely during bonding with etch-and-rinse as well as self-etch adhesives. The phenomenon is manifested as water blisters and water trees on the surface of the polymerized adhesive^28^,^29^; such slow fluid movement does not result in post-operative hypersensitivity^28^. Indeed, in *in vivo* testing of the augmented pressure adhesive displacement strategy with the use of a canine model, no aspiration of odontoblast nuclei was observed in stained histological sections of extracted teeth, when acid-etched, adhesive-coated test cavities were blown with augmented air pressure up to the strength of 0.4 MPa for 5 sec to displace adhesives into dentinal tubules (Chen *et al.*, unpublished results). Nevertheless, the use of augmented pressure to displace adhesives further down the dentinal tubules creates a rapid inward fluid shift. It has been shown that stimuli such as cold, which produce rapid outward fluid flow, generate more rapid and greater pulpal nerve responses than stimuli such as heat, which cause a rapid inward fluid flow^30^. Thus, it is not known whether the clinical use of the augmented pressure adhesive displacement strategy will create enough positive pressure to elicit transient episodes of post-operative hypersensitivity^31^. Further neurophysiology animal studies are required to understand the intensity of nerve impulses evoked by the use of this comparatively more aggressive adhesive displacement strategy. Additional human clinical studies should be conducted to evaluate the experience of post-operative hypersensitivity associated with the use of the augmented pressure adhesive displacement strategy.

Another issue to be highlighted is the potential cytotoxicity of dentin adhesives on vital dental pulps. Although adhesives are not applied directly over exposed dental pulps as in direct pulp capping, which has been shown to be an unacceptable treatment regime in many *in vivo* studies^32^,^33^,^34^,^35^, the augmented pressure adhesive displacement strategy may potentially introduce more unpolymerized adhesive resin monomers into the dental pulp when it is used in vital teeth with small remaining dentin thickness. Nevertheless, the use of etch-and-rinse adhesives in deep vital dentin with small remaining dentin thickness is a compromising issue from an endodontic point of view, and the challenges are not restricted to the intensity of adhesive displacement alone. In these situations, bonding may be more satisfactorily performed using a self-etch approach^36^.

Notwithstanding these potential limitations, the results of three-factor ANOVA in the present study indicate that the depth of the artificial carious lesions was significantly affected by the “intensity of adhesive displacement” and the “adhesive type”. Hence, the first null hypothesis that “the intensity of air pressure applied for displacement of adhesives applied to moist acid-etched dentin has no effect on the ability of the resin-dentin interface to resist demineralization by a single-species acidogenic bacteria biofilm” has to be rejected. Likewise, the second null hypothesis that “the use of an antibacterial adhesive has no effect on the ability of the resin-dentin interface to resist demineralization by the single-species acidogenic bacteria biofilm” also has to be rejected. It should be mentioned that the effect of “artificial caries induction time” is also statistically significant. For the three artificial caries induction times that produced carious lesions recognizable at the CLSM level, significant differences in lesion depths were predominantly discerned only after 25 days of artificial caries induction. This implies that future *in vivo* studies on the clinical efficacies of the two potential caries resistance improving strategies should aim at examination of long-term results for any perceivable advantages to be recognized. With regard to the interactions among the three factors, the interaction between “adhesive type” and the “artificial caries induction time” was statistically significant, while the interaction between the “intensity of adhesive displacement” and the “artificial caries induction time” was not statistically significant. The corollary interpretation of these statistical implications is that caries resistance can be potentially enhanced with the use of the experimental antibacterial adhesive when the augmented pressure adhesive displacement strategy is not employed. On the contrary, the contribution from the experimental antibacterial adhesive becomes less critical with the adoption of the augmented pressure adhesive displacement strategy. This rationalization is supported by one-factor ANOVA of the data derived from 25 days of artificial caries induction, in that no significant difference was observed between the A-G and the A-H group (Fig. 4, last two columns). In the pairwise comparisons that accompanied the use of this one-factor ANOVA, significant difference was only detected between the N-G group and the A-H group. That is, under the condition of the most severe artificial caries induction at 25 days, significant improvement in caries resistance could only be identified from augmented pressure adhesive displacement strategy of the experimental antibacterial adhesive. Thus, the third null hypothesis that “the combined use of augmented pressure adhesive displacement and antibacterial adhesive does not produce better caries resistance when compared with the separate use of those modalities” has to be rejected.

In the present study, CLSM was used to evaluate the development of secondary caries based on the principle of autofluorescence, without the use of supplemental fluorophores. The use of exogenous fluorescent markers in CLSM of the carious and non-carious resin-dentin interfaces is potentially misleading unless those fluorophores are chemically-conjugated to the adhesive or resin components; otherwise information only reveals the location of the fluorophores and not the location of the resin components to which fluorophores are added. Autofluorescence, by contrast, is common in biological tissues derived from plant and animals tissues, with potential biologic implications and diagnostic applications^37^,^38^. It refers to the natural emission of light biologic structures when they have absorbed light. The extracellular matrices of animal tissues contribute to autofluorescence emissions because of the intrinsically high quantum yield of collagen and elastin^38^,^39^. With respect to dentin, van der Veen and ten Bosch were the first to report demineralization-induced autofluorescence emission in CLSM imaging of sound and *in vitro* demineralized, non-carious human root dentin^40^. The implication of the early work by these authors is that intrafibrillar and/or extrafibrillar apatite possesses fluorescence quenching capability by deactivating the electronic excitability of the collagen in its excited state. Thus, removal of apatite from mineralized collagen will result in augmented autofluorescence of the demineralized collagen matrix. Banerjee and colleagues have further extended the use of autofluorescence emitted by demineralized dentin as an alternative marker for identification of carious dentin with CLSM^16^,^17^. When excited at 488 nm, emission intensity of carious dentin was reported to be 2.44 times stronger than that of mineralize sound dentin, while the peak of emission intensity spectrum was red-shifted from 545–556 nm^19^. While the increase in intensity of autofluorescence in carious dentin has been attributed to the breakdown of structural proteins and/or incorporation of protoporphyrins derived from bacterial metabolites^20^, recent studies from non-dental studies also indicate that increase in autofluorescence intensity may be related to alterations (reductions) in collagen crosslinks^41^,^42^.

Although the depth of the artificial carious lesion along the resin-dentin interface may vary in the four groups within one designated period of artificial caries induction, the thickness of the surface layer of demineralized dentin should not exhibit too much variation because they are created by the same single-species acidogenic biofilms. However, careful examination of the CLSM images of slices collected after the three periods of artificial caries induction showed that the surface layer of demineralized dentin was thicker in the N-G and N-H groups, and thinner in the A-G and A-H groups. The authors speculate that this observation is indicative of the possibility of diffusion of non-copolymerized antibacterial MAE-DB resin monomer from the adhesive polymer network toward the dentin surface, resulting in additional releasing-kill of the biofilm instead of contact-killing alone along the cavosurface margin^43^. The experimental antibacterial adhesive was formulated by arbitrarily adding 10 wt% MAE-DB to Single Bond 2. Thus, it is possible that the concentration of the MAE-DB in the antibacterial adhesive had not been optimized, and is in excess, resulting in leaching of the antibacterial resin monomer. Further studies should be performed with experimental antibacterial adhesives containing different concentrations of MAE-DB to limit its diffusional release, in order to avoid development of bacterial resistance due to the release of antibacterial species below their median lethal dose (LD_50_). This endeavor will result in the development of an antibacterial adhesive version with a high therapeutic index eliminate unnecessary toxicity to the host^44^.

Biomineralization is a process in which water from the intrafibrillar compartments of collagen fibrils are progressively dehydrated and replaced by apatite crystallites^45^,^46^. Because mineralization results in quenching of the intrinsic autofluorescence emitted by native collagen, it was originally anticipated that infiltration of collagen fibrils in acid-etched dentin by adhesive resin monomers would likewise result in quenching of the autofluorescence. However, the reverse is true in our experimental results. This prompted the authors to peruse further the intensities of autofluorescence emitted by different components of the non-carious resin-dentin interface. From the results of Fig. 5, demineralized dentin without adhesive infiltration emitted a higher intensity of autofluorescence than mineralized dentin, confirming the conclusions from previous work^19^,^20^,^40^. The adhesive employed also emitted faint autofluorescence, which, when infiltrated into the demineralized dentin, results in emittance of stronger autofluorescence by the hybrid layer. The results suggest that when applied to moist-acid-etched dentin, the adhesive could not quench the intrinsic autofluorescence of the demineralized collagen matrix. While it is preliminary to reach definitive conclusions based on the aforementioned observations, such a phenomenon may be indicative of incomplete replacement of the water present in the intrafibrillar compartments of completely demineralized dentin collagen (52 wt%) following the dissolution of the mineral phase^47^. Whether water can completely replace the pre-etched mineral volume in dentin collagen matrices have far-reaching consequences in the durability of resin-dentin bonds. This issue is still currently under intense debate. Whereas Bertassoni *et al.*^48^ critiqued that small resin monomers such as triethyleneglycol dimethacrylate are incapable of occupying the intermolecular spaces between collagen molecules, Takahashi *et al.*^49^ demonstrated that demineralized collagen fibrils behave as molecular sieves, enabling replacement of the intrafibrillar water by resin monomers in a size-dependent manner. Molecules larger than 10 kDa are excluded from collagen water while molecules smaller than 1 kDa can diffuse freely into the intrafibrillar compartments of collagen fibrils. It would be interesting in future studies to select an adhesive without intrinsic autofluorescence to determine if it can quench the autofluorescence of demineralized dentin after infiltration into the collagen matrix. If similar increased autofluorescence is produced, this would indicate that the adhesive does not completely infiltrate the intrafibrillar water compartments after the loss of the mineral component from the collagen fibrils.

Although the discovery of intense autofluorescence emitting from the resin-dentin interface of non-carious dentin brings up new challenges, it also opens the door to new non-invasive diagnostic opportunities. Autofluorescence emitted by human tissues (e.g. the tonsil) has been utilized in optical coherence tomography to visualize the tissues in a resolution that is higher than those derived from computed tomography, magnetic resonance imaging and ultrasound^50^,^51^. Application of optical coherence tomography in restorative dentistry is currently in its infancy of development, but has been shown to be an attractive non-invasive, interferometric cross-sectional imaging technique for identification of microgaps and demineralization progress around resin composite restorations^52^. Such a technique has the capability to be used for *in vivo* diagnosis^53^. It may be possible to combine the use of autofluorescence and optical coherence tomography in a single system to provide diagnostic information on the subsurface integrity of clinical resin-based restorations; research in this arena is in order.

## Additional Information

**How to cite this article**: Zhou, W. *et al.* Improved secondary caries resistance via augmented pressure displacement of antibacterial adhesive. *Sci. Rep.* **6**, 22269; doi: 10.1038/srep22269 (2016).

## Supplementary Material

Supplementary Information

## Figures and Tables

**Table 1:** 

Group (n = 15)	Air pressure	Adhesive	Time used for creating artificial caries
N-G	0.1 MPa	Single Bond 2	4, 8, 15, 20, 25 days
N-H	0.3 MPa	Single Bond 2	4, 8, 15, 20, 25 days
A-G	0.1 MPa	Experimental antibacterial adhesive	4, 8, 15, 20, 25 days
A-H	0.3 MPa	Experimental antibacterial adhesive	4, 8, 15, 20, 25 days

Grouping methods. N-G: commercial non-antibacterial adhesive + gentle adhesive displacement; N-H: commercial non-antibacterial adhesive + augmented pressure adhesive displacement; A-G: experimental antibacterial adhesive + gentle adhesive displacement; A-H: experimental antibacterial adhesive + augmented pressure adhesive displacement.

Application method: Applied etching agent for 15 sec, rinsed for 15 sec and dried gently for 2 sec, leaving the acid-etched dentin moist. Applied adhesive for 10 sec and dried for 5 sec using 0.1 MPa air-blow for adhesive displacement in G groups or 0.3 MPa air-blow for adhesive displacement in H groups at the distance of 1 cm from the bonding surface. Adhesive application and air-blowing were repeated, followed by light-curing for 10 sec. Cavities were subsequently restored with Z250 resin composite and light-cured for 40 sec according to the manufacturer’s instructions.
