# Supplementary material for: Improved secondary caries resistance via augmented pressure displacement of antibacterial adhesive
Source: Sci Rep. 2016 Mar 1;6:22269. doi: 10.1038/srep22269 (PMC4772086; doi:10.1038/srep22269)
Supplement: Supplementary Information [file srep22269-s1.pdf]

Supplementary Information

Improved secondary caries resistance via augmented pressure displacement of antibacterial adhesive

Wei Zhou[a,†], Li-na Niu[a,†], Li Huang[b], Ming Fang[a], Gang Chang[a], Li-juan Shen[a], Franklin R. Tay[c,]*, Ji-hua Chen[a,]*

Table of Content

S1 Energy dispersive X-ray map-scans of a representative artificial caries in N-G group after 20 days induction
S2 CLSM and SEM images of representative bonding interfaces after 8 days of artificial caries induction
S3 CLSM and SEM images of representative bonding interfaces after 15 days of artificial caries induction
S4 CLSM and SEM images of representative bonding interfaces after 25 days of artificial caries induction

Supplementary S1

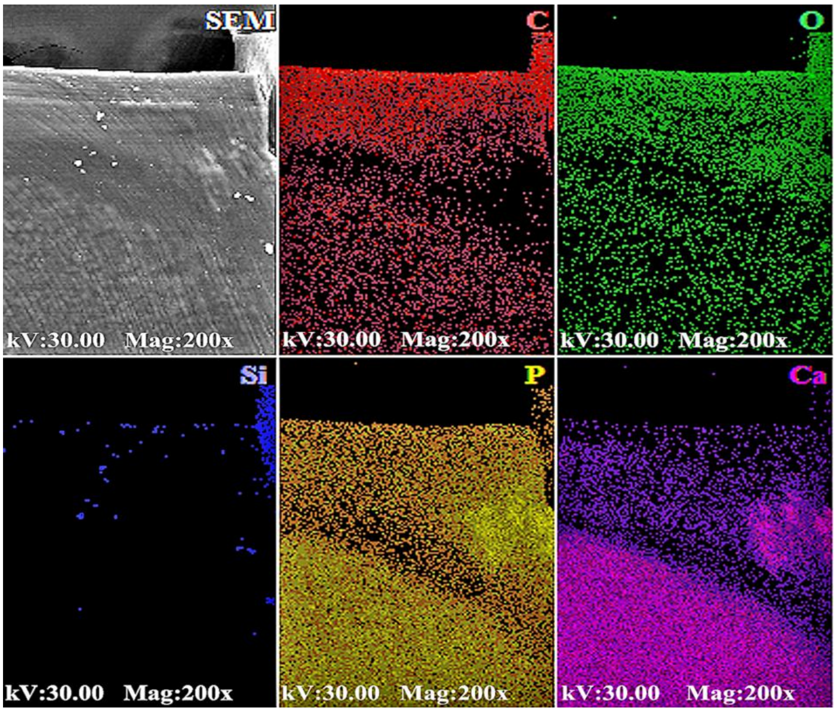

Figure S1 Energy dispersive X-ray map-scans of the elemental distribution from the top to the bottom of a wedge-shaped biofilm-induced artificial carious lesion in N-G group after 20 days induction. C: carbon; O: oxygen; Si: silicon; P: phosphorus; Ca: calcium.

Supplementary S2

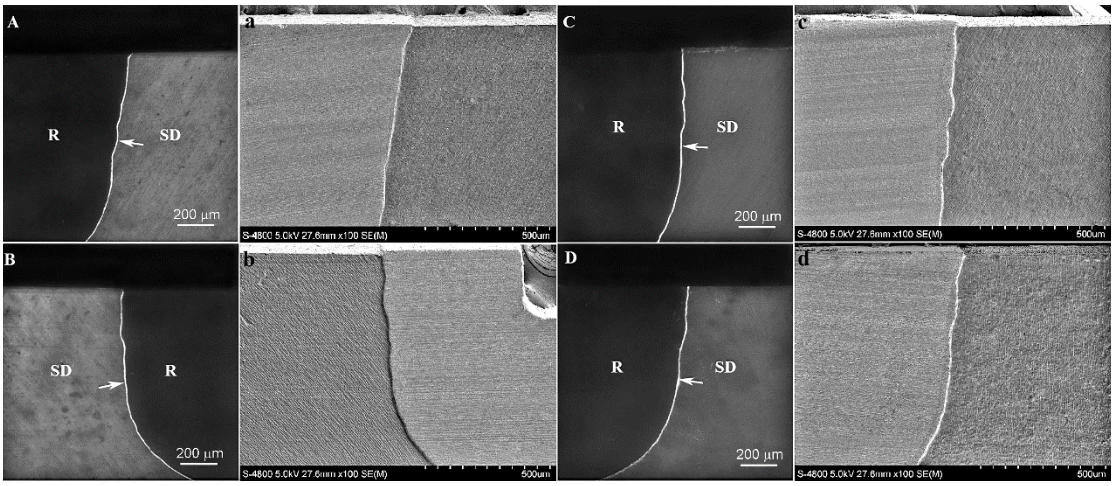

Figure S2 CLSM and supportive SEM images of representative bonding interfaces derived from the different groups after 8 days of artificial caries induction. (A) CLSM image of N-G group; (a) SEM image of N-G group; (B) CLSM image of N-H group; (b) SEM image of N-H group; (C) CLSM image of A-G group; (c) SEM image of A-G group; (D) CLSM image of A-H group; (d) SEM image of A-H group. No dentin surface demineralization or artificial caries could be identified from the restorative margin in both CLSM and SEM for all groups, as indicated by the absence of autofluorescence along the dentin surface. The non-carious resin-dentin interface exhibited intense autofluorescence and was continuous from surface to bottom of the restorative interface (arrows). The resin composite (R) did not emit autofluorescence, while faint autofluorescence was emitted by the mineralized sound dentin (SD).

Supplementary S3

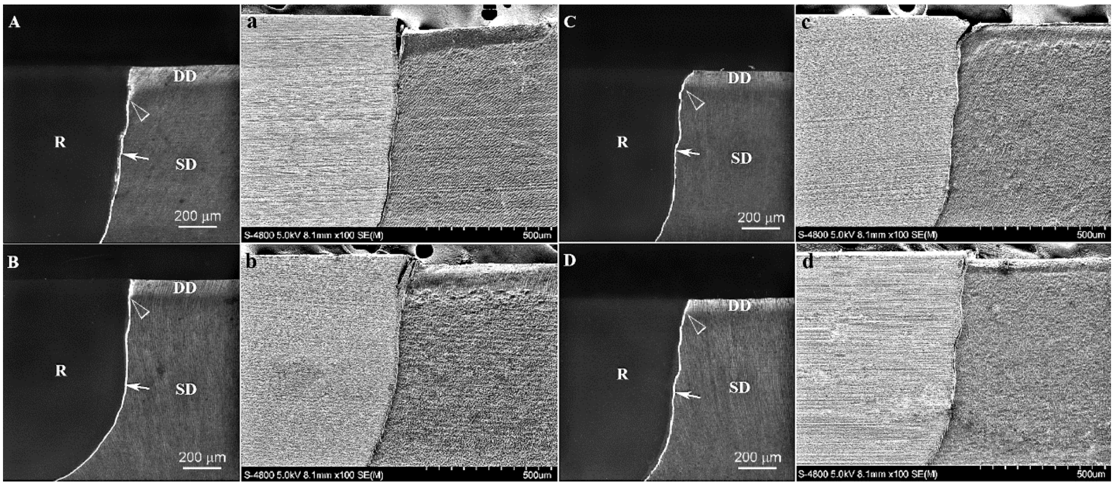

Figure S3 CLSM and supportive SEM images of representative bonding interfaces derived from the different groups after 15 days of artificial caries induction. (A) CLSM image of N-G group; (a) SEM image of N-G group; (B) CLSM image of N-H group; (b) SEM image of N-H group; (C) CLSM image of A-G group; (c) SEM image of A-G group; (D) CLSM image of A-H group; (d) SEM image of A-H group. Similar to specimens obtained from all the other caries induction time periods, intense autofluorescence was emitted by the resin-dentin interface (arrows) but not from the resin composite (R). Faint autofluorescence was emitted by the mineralized sound dentin (SD). Demineralization of dentin by acidogenic plaque biofilm can be identified as a layer of autofluorescence along the dentin surface (DD) in the CLSM images. Artificial caries formation can be recognized as a continuation of the autofluorescence along the superficial part of the resin-dentin interface (open arrowheads). Unlike CLSM imaging, features of dehydration shrinkage of the demineralized dentin surface collage and crack formation along the cavosurface margin could be seen in all SEM specimens.

Supplementary S4

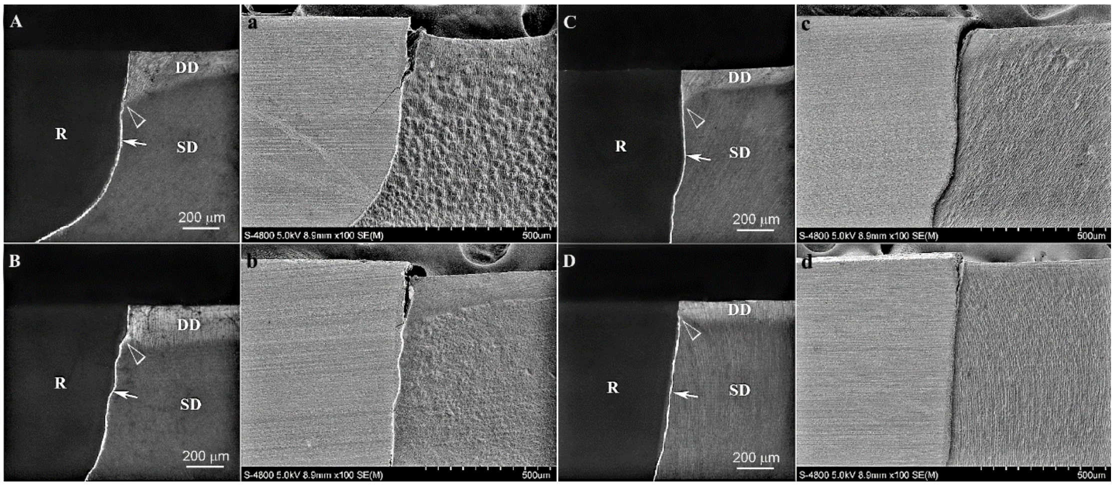

Figure S4 CLSM and supportive SEM images of representative bonding interfaces derived from the different groups after 25 days of artificial caries induction. (A) CLSM image of N-G group; (a) SEM image of N-G group; (B) CLSM image of N-H group; (b) SEM image of N-H group; (C) CLSM image of A-G group; (c) SEM image of A-G group; (D) CLSM image of A-H group; (d) SEM image of A-H group. Similar features (i.e. dentin surface demineralization (DD) or artificial caries along the restorative margin (open arrowheads)) were observed in all species derived from the different groups after 20 days of artificial caries induction (Figure 3), with variations in the thickness of dentin surface demineralisation and depth of artificial caries formation.
